# Neurologic Sequelae Associated with Hypertensive Disorders of Pregnancy

**DOI:** 10.3390/children8110945

**Published:** 2021-10-20

**Authors:** Mark S. Scher

**Affiliations:** 1 Pediatrics and Neurology, Rainbow Babies and Children’s Hospital, Case Western Reserve University School of Medicine, Cleveland, OH 44106, USA; mark.s.scher@gmail.com; 2 Department of Pediatrics, Division of Pediatric Neurology Fetal/Neonatal Neurology Program, University Hospitals Cleveland Medical Center, Cleveland, OH 44106, USA

**Keywords:** hypertensive disorders of pregnancy, maternal immune activation, the great obstetrical syndromes, developmental neuroplasticity, fetal/neonatal neurology, developmental origins/life-course theories, global burden of disease

## Abstract

Hypertensive disorders of pregnancy (HDP) contribute to adverse gene-environment interactions prior to conception and continue throughout pregnancy. Embryonic/fetal brain disorders occur from interactions between genetic susceptibilities interacting with acquired diseases or conditions affecting the maternal/placental fetal (MPF) triad. Trimester-specific pathophysiological mechanisms, such as maternal immune activation and ischemic placental syndrome, contribute to adverse peripartum, neonatal and childhood outcomes. Two diagnostic approaches provide timelier diagnoses over the first 1000 days from conception until two years of age. Horizontal analyses assess the maturation of the triad, neonate and child. Vertical analyses consider systems-biology from genetic, molecular, cellular, tissue through organ networks during each developmental niche. Disease expressions associated with HDP have cumulative adverse effects across the lifespan when subjected to subsequent adverse events. Critical/sensitive periods of developmental neuroplasticity over the first 1000 days are more likely to result in permanent sequelae. Novel diagnostic approaches, beginning during pre-conception, will facilitate the development of effective preventive, rescue and reparative neurotherapeutic strategies in response to HDP-related trimester-specific disease pathways. Public health policies require the inclusion of women’s health advocacy during and beyond their reproductive years to reduce sequelae experienced by mothers and their offspring. A lower global burden of neurologic disease from HDP will benefit future generations.

## 1. Introduction

**Figure 1:**
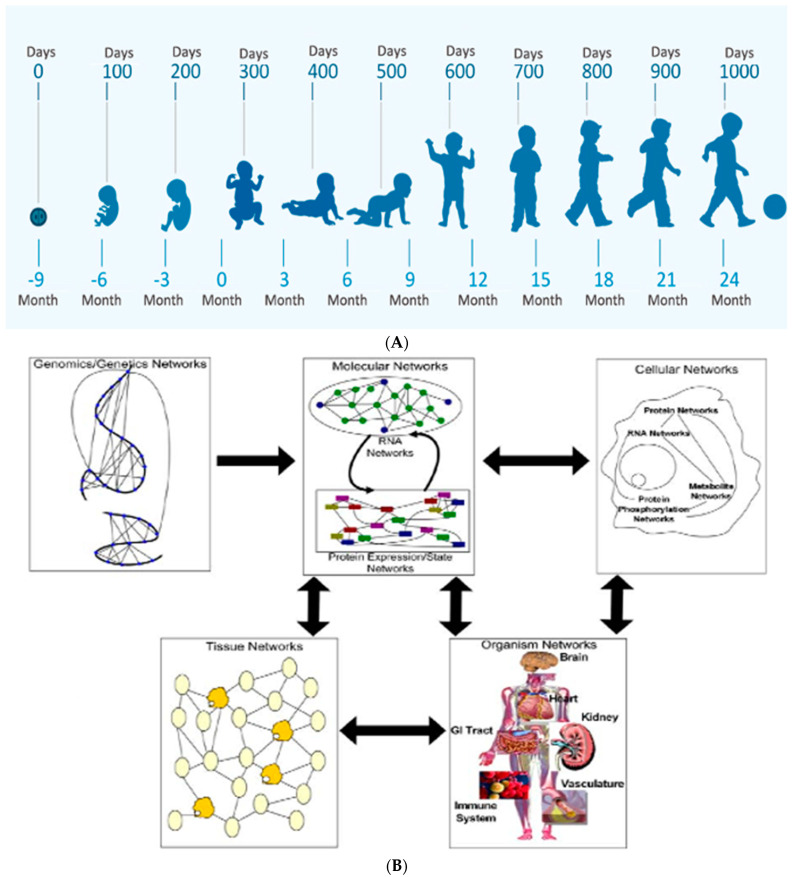
The horizontal/vertical diagnostic process across maturing phenotypes during the first 1000 days: (**A**) This horizontal perspective over the first 1000 days starts at conception through two years of age from embryo to the young child, as depicted by this WHO figure. (**B**) The flow of information across biological systems through a hierarchy of networks depicts the vertical diagnostic process. Successive panels highlight the interactions between different networks that comprise a biological system. Genomic networks depict interactions between DNA sequences which may result in a longer range, as well as more short-term changes, that alter chromosome structures to modulate gene activity These interactions also promote synergistic effects on higher-order phenotypes. Genomics networks in turn subserve molecular networks comprised of RNA, protein, metabolites, and other molecules within the system. Molecular networks are components within cellular networks in which complex interactions between these networks result in the complex phenotypes that define living systems. Tissue networks comprise cellular networks which are influenced by molecular and genomics networks. Organism networks then comprise tissue networks that define the component cellular and molecular networks. Disease states are complex phenotypes that emerge from this complex web of interacting networks, given disrupted genetic and environmental interactions to the living system (Sieberts 2007).

Two diagnostic approaches assist in the prediction of brain health or disease throughout the life-span. [1] Over the first 1000 days, assessment of a patient’s form and function is applied to the horizontal analytic approach, starting before conception and continuing across three trimesters of pregnancy (Figure 1A). The maternal/placental/fetal (MPF) triad is the “patient” who experiences trimester-specific maturational changes to favorable or adverse conditions. The MPF triad interactions influence the developing embryonic/fetal brain in relation to other fetal systems such as gestational age advances (Figure 1B) [2]. The vertical diagnostic approach considers genomics through multi-systemic interplay during each developmental interval. These interactions begin before conception to influence interrelated mechanisms, altering the developing fetal nervous system within each triad, followed by the neonate and child. Adaptive or maladaptive consequences in response to on-going gene/environment (G × E) interactions to diseases and adversities continue across the lifespan.

Both diagnostic approaches allow the fetal/neonatal neurologist (FNN) to anticipate adverse outcomes from any disease process that may potentially affect the developing nervous system across time-periods. This analytic process can be applied to pathophysiological mechanisms associated with specific categories of disease affecting the MPF triad, such as from hypertensive disorders of pregnancy (HDP). Brain health or disease experienced after HDP during critical/sensitive developmental time-periods over the first 1000 days will more likely remain permanent across the lifespan. Novel diagnostic approaches, starting during pre-conception are required that potentially can be applied to all levels of maternal care across the three trimesters. These advances will lead to effective and timelier neurotherapeutic interventions that combine preventive, rescue and reparative strategies for the developing brain in response to the diseases and adversities [3] associated with HDP. Both diagnostic approaches will later assist in on-going analytic perspectives for neurologists throughout childhood and into adulthood.

This analytic approach requires a working knowledge by an interdisciplinary team of practitioners regarding the maturing embryonic/fetal brain within the MPF triad across three trimesters in association with HDP. Such an approach will better select the appropriate diagnostic tests and treatment interventions that are patient-centric from a developmental perspective regarding a vulnerable MPF triad.

The practitioner is confronted with a spectrum of HDP disorders in women, including hypertensive diseases before conception, gestational hypertension, early and late preeclampsia, HELLP syndrome and eclampsia. For this discussion of potential sequelae after HDP, no single diagnostic test or therapeutic intervention will be effective for all women at all times before and during pregnancy. New obstetrical research will offer the clinician with different options and timing that can be standardized for maximal effectiveness, beginning prior to conception. This will be discussed in Section 10.

HDP affects 5–7% of the world population of women and their offspring. In one recent study, as many as 7–15% of the world’s population of women will experience HDP when assessed on a per-woman basis [4]. Given such a potentially large proportion of affected females, adverse outcomes must be anticipated for women during and beyond their reproductive years [5] as well as for their offspring [6]. Associations with specific outcomes such as autistic spectrum disorders (ASD) and attention deficit hyperactivity disorder (ADHD) will be included in this discussion, representing multiple forms of clinical expression of neurologic disorders that may present in the same individual. Critiques of outcome studies must consider limitations based on study design. When considering ASD and ADHD, for example, compared with intellectual disability and general cognitive performance [7], the latter are more likely confounded by shared familial factors that require consideration of G × E, beginning during pre- conception. Population-based studies reporting adverse outcomes after HDP also lack accurate definitions of ASD and ADHD, resulting in underestimates of the percentage and severity of outcome.

Improved diagnostic algorithms of HDP will help reduce the global burden of neurologic disease. As discussed at the conclusion of this review, this should be a public health priority for women and their offspring given the high prevalence of HDP. International efforts, such as the sustainable development goals proposed by the WHO [8,9], must be applicable to women and children both in resource-rich and poor nations. Transgenerational diseases affected by HDP are specific to a nation’s or a region’s healthcare and with the socioeconomic and cultural conditions that will influence outcome [10]. This specific discussion addresses why neurologic sequelae occurs in persons susceptible to adverse effects from HDP across the lifespan. While adverse multi-system maternal effects, such as impaired cardiorespiratory and metabolic health (i.e., the metabolic syndrome), are also important to recognize, childhood and adulthood brain disorders will be the focus of this discussion [11].

## 2. Gene/Environment Interactions Influence Clinical Expression

Transgenerational followed by early pregnancy G × E influences subject the MPF triad to HDP, with different expressions of neurologic sequelae by the offspring. Pre-conceptional genetic susceptibilities have been identified through investigations such as high-throughput genome-wide association studies (GWAS) and epigenetic studies [12]. Improved diagnostic testing advances in the future will apply knowledge of the pathophysiological basis to evidenced-based treatment options for the maximal beneficial outcome. Inherited genetic changes prior to pregnancy may be then followed by de novo genetic alterations with conception expressed by the embryo and placenta. Single and multiple gene mutations during miosis are made more complex by mechanisms such as imprinting and germ-line mosaicism. Post-mitotic epigenetic and somatic mosaicism mechanisms further contribute to genetic diversity expressed by more diverse phenotypes. This continuum of the pathologic process potentially results in more severe disorders from HDP across three trimesters of pregnancy. Such genetic aberrations impair both placental and fetal brain structure and functions within the developing MPF triad. 

Candidate genes and pathways responsible for sequelae are more accurately identified by integrated bioinformational analysis [13]. Differing sets of genetic and epigenetic events lead to alteration of gene expression at the transcriptional or translational levels, involving multiple functional signaling pathways responsible for placental and fetal brain function. These analyses have been initially applied to study adverse effects from late-onset PE. Up-regulated gene expressions were associated with extra-cellular matrix organization, while down-regulated genes were alternatively identified with the immune process. These studies considered the imbalance of angiogenesis and hypoxia on placental and fetal brain after late-onset PE. This approach needs to be expanded to include the study of potential genes and pathways in the pre-conception period and pre-functional placenta before eight weeks post-conception to address pre-conceptional hypertension, gestational hypertension and early-onset PE that potentially disrupt implantation and early placentation. More profound adverse effects on the developing embryonic/fetal brain [14] may result given vulnerability during earlier stages of development. 

Co-morbidities associated with maternal diseases and adversities further augment genetic/epigenetic effects [15] beginning before conception. Both infectious and non-infectious inflammatory states potentially alter the MPF triad when exposed to HDP. Pregnancy-related infections, maternal diabetes, autoimmune disorders, maternal obesity and toxic stresses can be synergistic in the same individual, worsening the adverse effects from HDP as pregnancy advances. Identification of proteomic biomarkers representing genes and related pathways before and during early pregnancy offer the opportunity to develop screening protocols to treat specific forms of HDP, as well as co-morbidities [16]. Depending on the burden of transgenerational heritability, varying degrees of sequalae may subsequently be expressed across generations. Pre-clinical testing in women and girls should begin as they enter their reproductive years.

Cross-species expression of the MPF triad structure and function suggests preserved evolutionary biological processes, with either beneficial and adverse outcomes in humans [17]. This transmission plays a key role in determining human neurodevelopment across generations, worsened by biosocial disadvantages [18]. This concept is applicable to outcome research related to HDP when designing more effective time-sensitive diagnostic and therapeutic options.

Structural markers detected by fetal surveillance using sonography and more detailed fetal neuroimaging with magnetic resonance imaging include a wider range of detectable brain lesions, including major malformations with or without lethality to region-specific lesions represented by focal developmental anomalies and destructive lesions. The form and severity of these anatomical biomarkers depend on the trimester-specific timing and specific pathophysiological pathways that were activated by G × E interactions associated with HDP as well as other diseases entities with similar harmful effects from hypoxia/ischemia, inflammation and coagulopathy. Early pregnancy anomalies range in severity from neural tube defects, holoprosencephaly and schizencephaly to focal cortical dysplasias. During the latter half or pregnancy, destructive lesions predominate and may accompany earlier anomalous lesions associated with HDP-related effects, such as the maturing MPF triad experiences in on-going disease.

G × E interactions result in different neurologic sequalae specific to pre-conception and the trimester-specific time-periods when the MPF triad is affected by HDP. Primary prenatal neurologic structural/functional disorders without systemic involvement may occur. Secondary brain lesions alternatively result from multi-systemic conditions that also contribute to adverse neurologic outcomes. Disease processes, such as maternal immune activation [19] and ischemic placental disease [20], correlate with developmental anomalies or destructive lesions of the developing brain, dependent on the trimester during which the disease predominated. Multiple fetal organ systems may also be affected, contributing to more severe adverse effects on the fetal brain by altered hypoxic-ischemic, inflammatory or other disease pathways.

G × E interactions associated with HDP continue to be expressed after birth as the great neonatal neurological syndromes (GNNS) of encephalopathy (NE), seizures (NS), stroke (NSK) or encephalopathy of prematurity (EP) [1]. The severity of these neonatal disease-phenotypes reflects prenatal MPF triad conditions or disease states superimposed on peripartum and postnatal medical complications. Adverse outcomes will also depend on gestational maturity, with comparatively greater injury to the preterm versus the full-term brain after hypoxic/ischemic, infectious and non-infectious inflammatory, and hemostatic processes expressed as multi-systemic disorders.

Pediatric infections, trauma and multi-systemic diseases later in childhood may further worsen sequelae after HDP, stressing the continuity of risk from G × E interactions during postnatal maturation. Both communicable and noncommunicable childhood diseases contribute to adverse outcomes, involving hypoxic/ischemic, inflammatory and hemostatic disease pathways. Biosocial adversities affecting the child within the family and community contribute to the extent and complexity of sequelae. Developmental disorders, such as intellectual disability, autism and cerebral palsy, as well as behavioral/cognitive deficits and epilepsies, are potential sequalae expressed throughout childhood and adolescence. Each disorder may either present independently or as co-morbidities. Associations with HDP need to be considered.

Sequalae later present with maturation into adulthood. The aging brain’s pathophysiological mechanisms will result in long-term responses to the prenatal conditions of HDP, as well as to other adverse diseases or conditions affecting the MPF triad. Diverse sequalae include cerebrovascular diseases, dementias, adult-onset epilepsy and neurodegenerative disorders. G × E interactions at the end of the lifespan are significantly influenced by permanent maladaptive developmental neuroplasticity that occurred during the first 1000 days.

## 3. Early Pregnancy Effects on Embryonic/Fetal Brain

Complex MPF triad G × E interactions from HDP early during pregnancy profoundly impairs neuronal and glial progenitor cells within transitory brain structures [21]. Disruption of neurulation and segmentation/cleavage impair neural tube closure and midline brain structures, expressed as major brain malformations such as neural tube defects and the holoprosencephaly spectrum in the susceptible fetus. These anomalies may be documented by prenatal sonography and fetal magnetic resonance neuroimaging [22], or only after birth with neonatal or childhood studies [23,24]. Sensitivity and specificity of the abnormal images obtained will be limited by the current technologies available for clinical applications. Falsely negative neuroimages do not eliminate the possibility of abnormal connectivites below the resolution to detect.

**Figure 2:**
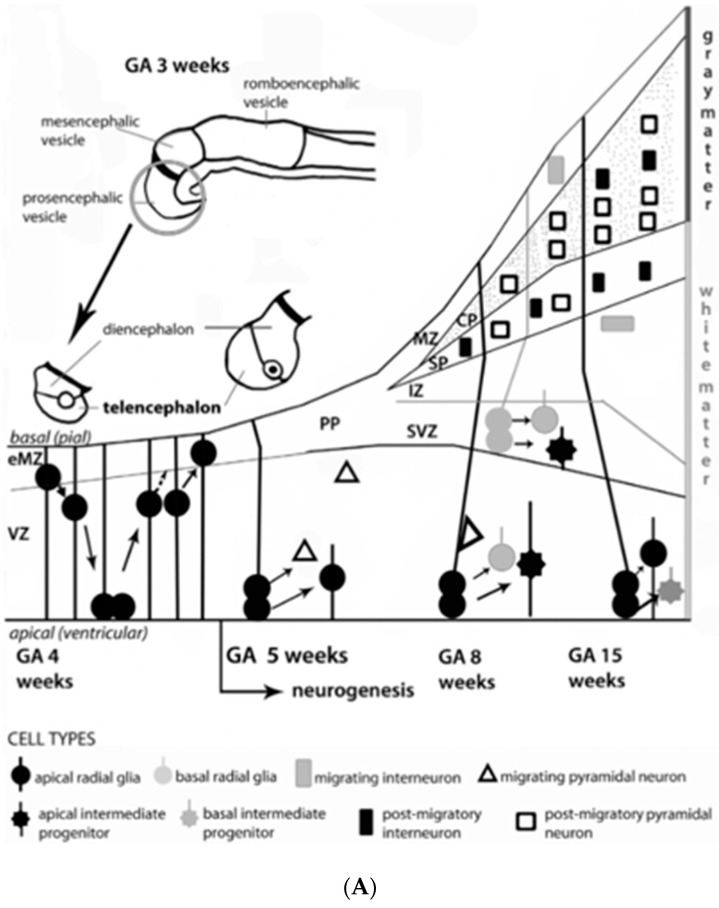

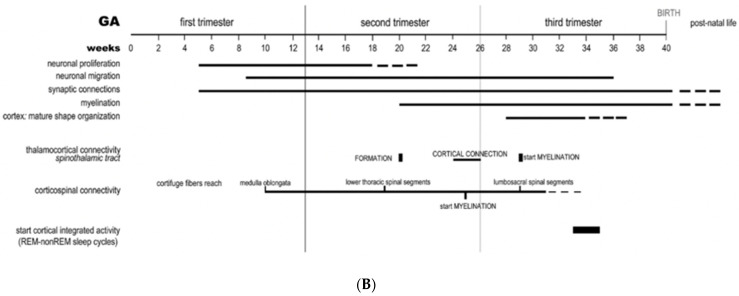
(**A**). Developmental brain processes begin during gestational age (GA) in weeks 2–3 with the formation of the neural tube. At GA week 4, the rostral segment of the neural tube forms the prosencephalic, mesencephalic and romboencephalic vesicles. The prosencephalic vesicle then forms two vesicles that later become the telencephalon and the diencephalon (thalamus, hypothalamus, and other structures). Telencephalic development is depicted in the schematic diagram published by Borsani et al., 2011. [21] Initially, the telencephalic primordium is comprised of proliferating and differentiating neuroepithelial cells (also termed neural stem cells), characterized by interkinetic nuclear migration to form the ventricular zone (VZ). Radial glial cells are particularly prominent and distinctive and undergo early exponential proliferation. Increasing numbers of progenitor/intermediate progenitor cells contribute to the thickness of VZ by week 4. Progenitor neurons include interneurons, which form early and migrate to the marginal (MZ) and intermediate zones (IZ). The MZ eventually forms cortical layer I before the cortical plate (CP) formation as an early MZ (eMZ). A cell-sparse IZ compartment exists before the CP. Radial glial cells and intermediate progenitor cells can be classified into two distinct subpopulations: apical neurons in the VZ with bipolar fibers, and basal types, which delaminate from VZ with unipolar basal fibers. Apical progenitor cells in the VZ and basal progenitor cells in the subventricular zone (SVZ) are considered to be the main source of pyramidal neurons. Around GA week 5, neurogenesis begins at week 5 with neuronal precursors proliferating rapidly within the VZ. The neurons within the first recognizable cortical layer, known as the pre-plate (PP), form the earliest synaptic connections. PP is a transient structure present before the appearance of the CP. Around week 7, the PP cells contribute to the subplate (SP), which remains below the CP after its formation and contains post-migratory pyramidal neurons and interneurons. Accumulation of basal progenitor cells create a distinct new compartment above the VZ, termed the SVZ, where intermediate progenitor cells proliferate. By GA week 8, radially migrating neurons from VZ and SVZ initiate the development of the layered CP forming from inside to outside. Pyramidal neurons eventually migrate outward along the radial glial cells [21]. These neuroembryonic changes influence subsequent brain development during the later trimesters (**B**). This timeline depicts neurobiological telecephalic processes with sensory/motor connectivities during human brain maturation. Successive weeks with increasing gestational age (GA) relate to the major events during fetal neural development: neuronal proliferation; neural migration; synaptic connections; myelination; cortex (mature shape organization); thalamocortical connectivity (spinothalamic tract); cortico-spinal connectivity; with the early expression of cortically-integrated activities such as REM-non-REM sleep cycles [21].

Multi-potential precursor populations undergo proliferation, differentiation and migration during the first twenty weeks within multiple transient structures [25], such as the ventricular/marginal zones, and the ganglionic eminence (Figure 2A, B). Impaired secondary yolk sac function during abnormal placentation may contribute to impair neuroembryonic structures during the first 8 weeks, even before the placental function begins. Altered signaling processes, such as Wnt pathways [26], promote abnormal patterning, which affect axon guidance, synapse formation and the neuronal connectivities required later in pregnancy. Multiple protein and pathway aberrations alter cell positions, neurotransmitter expressions and neuroplasticity responses [26,27,28] throughout the first half of pregnancy as affected by disease processes associated with HDP. Early pregnancy abnormal processes result in a range of adverse outcomes from diffuse/multifocal migrational anomalies to focal cortical dysplasias. Clinical expressions only appear later during the first 1000 days.

Altered progenitor cell populations within the neuroectoderm or yolk sac later promote abnormal connectivity during the second half of pregnancy with postnatal neurologic sequelae (Figure 2A, B). Altered interneuronal development represent one microglial progenitor population within transient brain structures that later form abnormal neuronal circuitry, given the importance of this cell population [28]. Comprising 25–30% of cortical populations in primates [29], abnormal interneuronal maturation later clinically express imbalanced excitatory/inhibitory properties (i.e., interneuronopathies), starting during the first 1000 days. Altered early transcriptomics [30], starting during the first half of pregnancy, are later expressed as pediatric neurodevelopmental disorders including intellectual disability and autism, as well as epilepsy [31].

Impaired brain development during the first half of pregnancy associated with HDP affects the interactions of multiple neuronal, glial, and angiogenic precursors, particularly within the developing neurovascular unit (i.e., blood–brain/blood–CSF barriers). Deficient pro-angiogenic growth factors, such as placental growth factor [32] and vascular endothelial growth factor [33], associated with the disease process associated with HDP may alter fetal cerebrovasculature with profound effects on neuronal/glial development. Developmental, cognitive/behavioral and epileptic disorders are later expressed across the life-span that began during the first 1000 days. Sex-specific disparity is one of multiple confounding factors that influence outcomes related to HDP [34].

Immune intolerance between the mother and embryo/fetus from abnormal placental implantation and development result in maternal immune activation (MIA), which later influences the expression of multiple psychiatric and neurological disorders over the life-span [19]. Specificity and severity of sequelae are determined by the degree of disruption by MIA, affecting the placental properties required for nutrient/waste functions as well as multiple growth factors that are important for the developing nervous system [31].

**Figure 3:**
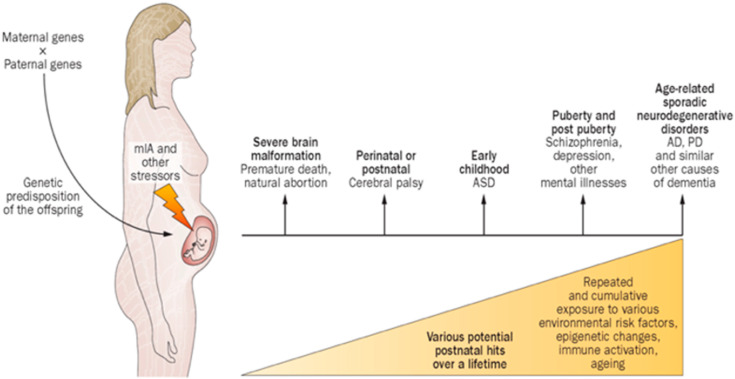
Proposed interrelated factors associated with maternal immune activation in humans, expressed as a spectrum of neurologic/behavioral disorders expressed by the child, juvenile, adult or elderly individuals. Abbreviations: AD, Alzheimer’s disease; ASD, autism spectrum disorder; MIA, maternal immune activation; PD, Parkinson’s disease [36].

Consideration of G × E interactions over the first 1000 days begins with an understanding of the vulnerability from MIA effects after infectious and non-infectious inflammatory conditions, particularly during early pregnancy. A broad range of diseases and conditions result in MIA, including HDP, and affect the maturing components of the MPF triad, with altered fetal brain connectivity. While prevalence estimates of MIA remain imprecise, a significant percentage of children and adults later express early and long-term neurologic disorders from maladaptive developmental neuroplasticity as a result of MIA associated with diseases such as those from HDP. Improved neurotherapeutic interventions need to be developed in order to apply an understanding of MIA (Figure 3) in relation to multiple disease states such as those from HDP. Adverse effects may be later expressed across the lifespan and constitute a major public health challenge. Policies need to recognize women’s healthcare during reproductive years regarding disease states such as those associated with HDP that will benefit their health into old age, as well as their male and female offspring [35].

## 4. Later Pregnancy Effects Associated with the Great Obstetrical Syndromes

Disordered cellular migration, dendritic arborization, synaptogenesis and myelination within the developing fetal brain is predominant during the latter half of pregnancy. These developmental fetal brain stages are particularly active within the maturing cortex and subplate zone [37]. Anomalous brain development that occurred during the first half of pregnancy, may then be superimposed on destructive brain lesions with diseases expressed during the second half of pregnancy (Figure 2A,B). Mechanisms associated with disease states such as HDP include diseases in the second and third trimester-specific hypoxic–ischemic, inflammatory, and hemostatic disease pathways [38,39].

**Figure 4:**
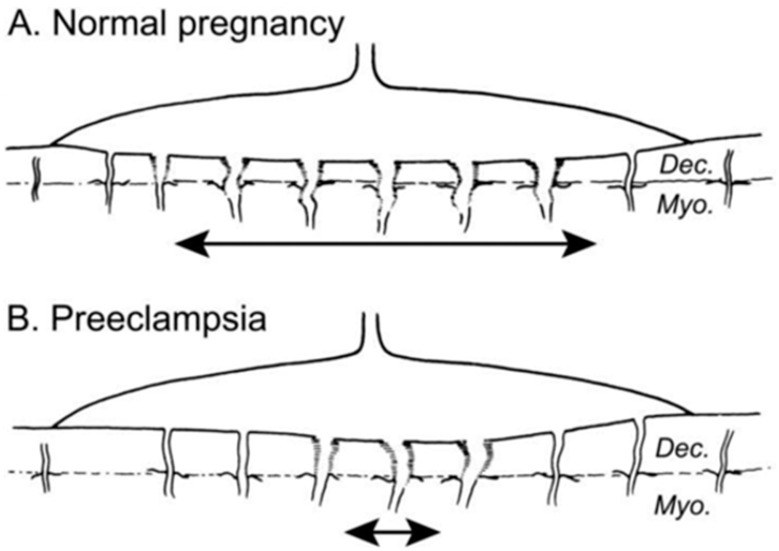
(**A**) Normal placental bed with full transformation of the myometrial spiral arteries with widened vascular diameters except at the periphery of the placental bed. (**B**) Defective deep placentation is characterized by non-transformation of the myometrial spiral arteries, reducing placental blood flow within the central area with a paucity of deep placentation [42].

Di Renzo introduced the term “the great obstetrical syndromes” (GOS) (2009), referring to abnormal maternal, placental and fetal outcomes as a result of abnormal placental angiogenesis. A growing list of adverse outcomes from GOS can affect the MPF triad. Phenotypes specific to each of the three triad components include preeclampsia, diabetes, morbidly adherent placenta, abruptio placenta, premature rupture of membranes, fetal growth restriction, fetal demise, and prematurity. All these conditions adversely affect MPF triad health, with impairment to fetal brain development specific to the developmental stage. Abnormal placental angiogenesis during the second half of pregnancy consists of incomplete spiral artery remodeling and shallow embedding (Figure 4A,B). This angiogenetic disorder was initiated during the first half of pregnancy through G × E interactions involving common disease pathways that impair trophoblastic development by immunological and apoptotic mechanisms. Altered leucocyte and macrophage functions during first-trimester implantation and embryonic placental development [40,41] are later expressed as ischemic placental syndromes (IPS), resulting from abnormal placental angiogenesis with adverse maternal/fetal outcomes represented by the GOS [20]. These adverse outcomes may be expressed as abnormal maternal/fetal clinical signs, such as the varying severities of maternal hypertension or abnormal surveillance test results, representing maternal/fetal malperfusion conditions during the latter half of pregnancy. 

HDP are expressions of abnormal placental angiogenesis associated with IPS [43]. Pathophysiological mechanisms with HDP combine asphyxial, inflammatory and hemostatic conditions that increase the risk of brain injuries throughout the second and third trimesters into the peripartum period. Specific placental lesions, such as maternal or fetal underperfusion, have been correlated with fetal demise, abnormal neonatal outcome and childhood neurological development in systematic reviews [44]. Immediate delivery versus expectant monitoring must be judged for each pregnancy when the clinical situation deteriorates to a degree that requires consideration of altered obstetrical management of the pregnancy to mitigate injuries to the mother and fetus. Preterm more than full term survivors subjected to HDP may be at an increased risk of further brain injuries secondary to adverse postnatal medical conditions requiring intensive care [45]. The preterm brain is more likely to be altered by maldevelopment and destructive changes during pregnancy as a result of HDP-related IPS.

## 5. Peripartum and Intrapartum Considerations

Adverse trimester-specific conditions from HDP can negatively impact peripartum events close to and including labor and delivery [46]. Obstetrical strategies may need to be re-adjusted during late pregnancy, based on gradual worsening trends or sudden events detected by abnormal fetal surveillance test results. These changes can be represented by abnormal anthropometric measures, biophysical scales, doppler-flow indices and fetal heart rate patterns. While fetal hypoxia is more often an adaptive response that protects against brain injury [47], loss of fetal well-being may also be associated with brain damage. Current fetal surveillance tests, however, are poor predictors of past or contemporaneous fetal brain injury. Peripartum abnormalities may reflect current physiologic changes associated with remote brain injuries that already occurred from HDP. Pre-existing brain anomalies or injuries may alternatively be present despite falsely negative test results. The obstetrical healthcare provider is presently unable to distinguish this subset from the majority of MPF triads who either remain protected or who have already been injured [1]. 

Adverse intrapartum events associated with HDP may only later be identified during neonatal or childhood time-periods. Whether sentinel or gradual in onset, presumed fetal distress detected during labor and delivery by surveillance testing is also a poor proxy that cannot reliably identify the presence or timing of brain injury. The peripheral chemoreflex remains primarily an adaptive response, activated during uterine contractions to protect against primarily brain, cardiac and adrenal injuries in most situations as the child descends the vaginal tract. Specific clinical scenarios can result in intrapartum fetal brain injury [48], particularly when associated with sentinel events. Abruptio placenta from morbidly-adherent placenta, umbilical cord anomalies such as prolapse, and uterine lesions such as rupture are three categories resulting in sudden (i.e., sentinel) hypoxia-ischemia stress [1]. However, antepartum with or without intrapartum MPF triad factors must be considered, which better explain sequelae. 

Complex disease conditions represented by HDP are best interpreted by applying horizontal and vertical analytic perspectives to anticipate risk or occurrence of brain injuries prior to, as well as during, labor and delivery. Interdisciplinary consensus reports periodically update the evolving scientific bases for these analyses [48,49]. Complex interactions over time from multiple etiologies associated with HDP may cumulatively contribute to brain lesions beyond current testing to detect or predict. Future trimester-specific testing tools that combine pre-conception and trimester-specific genetic biomarkers compared with serial volumetric/functional imaging studies of fetal brain and placenta as pregnancy advances will improve diagnostic accuracy [22,50], as discussed in Section 9.

## 6. The Great Neonatal Neurological Syndromes

Complex phenotypes after birth are expressed collectively as the great neonatal neurological syndromes (GNNS). Similar to the GOS, trimester-specific G × E interactions affect the MPF triad during postnatal life, expressed as the GNNS. Differences from GOS include: (1) clinical presentations include events specific to the peripartum and neonatal time-periods; (2) phenotypic expressions of GNNS are not reliably detected across trimesters for all maternal levels of care, particularly with seemingly low-risk pregnancies, (3) early and later pregnancy factors cumulatively affect MPF triad components closer to delivery expressed as the GNNS. NE, NS, NSK, and EP are the four major GNNS categories [1] that need to be considered as separate clinical expressions as well as overlapping categories in the same neonate.

Negative outcomes associated with the GNNS occur despite adjustments in obstetrical management closer to delivery to conditions associated with HDP. Introduction of resuscitative interventions and neurocritical care may not significantly alter antepartum adverse outcomes [1,3]. FNN applications of horizontal and vertical diagnostic perspectives improve evaluations for each neonate expressing a GNNS as additional information is acquired during the first postnatal hours to days when neurocritical care interventions are offered. 

Acute infections, metabolic-toxic and traumatic etiologies may best explain peripartum or neonatal-acquired encephalopathies and/or brain injuries expressed by the GNNS. Alternatively, trimester-specific MPF triad G × E interactions associated with HDP have a varying clinical expression during peripartum and neonatal time-periods, based on the timing and etiologies associated with brain anomalies or injuries superimposed on acute disease. Childhood neurologic disorders may later present, despite neonatal neurocritical care interventions for the GNNS as well as in the absence of early neonatal disease expression. 

Neonatal evaluations specific to each GNNS have been discussed in more detail elsewhere and are applicable to clinical assessment after HDP [1]. NE, NS, NK and EP may each present with depressed levels of arousal after birth, requiring resuscitation and intensive care without intrapartum contributions to brain injury. Despite presenting with these neonatal syndromes, these children may have experienced antepartum factors during first, second or early third trimesters prior to labor and delivery resulting in antepartum brain lesions. Many children with remote brain injury may later present with loss of fetal well-being during intrapartum FHR monitoring, given acute clinical deterioration associated with chronic uteroplacental disease [51]. Chronic placental malperfusion syndromes associated with HDP may later express NE, with or without NS, NSK or EP. For less than 10% of the full term neonatal population at risk for neurologic sequelae, intrapartum hypoxic-ischemic encephalopathy (HIE) was considered the presumed predominant cause of brain injury. This has been reported more frequently with preterm rather than full-term neonates [52] when associated with HDP, often associated with remote placental diseases resulting from MIA and IPS.

Paradoxically, lower mortalities and adverse neurological outcomes such as periventricular leukomalacia, intraventricular hemorrhage and cerebral palsy have been retrospectively reported in specific studies of extremely and very preterm infant populations when associated with HDP, compared with cohorts without HDP [53]. HDP may offer a protective effect for a subset of children who experienced HDP. System-biology mechanisms associated with advanced brain maturation or the promotion of anti-angiogenic factors may protect the fetal brain. However, limited follow-up, selection bias and multifactorial effects responsible for adverse outcomes may underestimate long-term sequelae, even in the absence of short-term injuries.

Diagnostic strategies to assess the GNNS requires a time-sensitive interpretation of serial examinations, neuroimaging, electroencephalography and blood/serum studies throughout the neonatal hospitalization [1]. Multi-systemic effects from HDP affect preterm more often than full term survivors [54]. While the interplay between multiple systems affecting the brain must be considered, discussion specifically regarding the sequelae affecting the nervous system highlights the present discussion.

One important diagnostic test regarding the timing and etiology of brain injuries should include gross and microscopic examinations of the placenta, cord and uterus. While the completed pathology report may not be available until after the first week of life, findings often more accurately estimate an antepartum time-period to diseases that precede events closer to delivery. Trimester-specific pathophysiological mechanisms may better explain different disease processes associated with HDP after pathological analyses of the placenta, cord and uterus. This explanation will better assist the family for both diagnostic accuracies, as well as prognosis and anticipatory care. Revised pathological classifications have strengthened these chronic associations [55]. However, limitations in the sensitivity and specificity of present pathological analyses include falsely negative placental/cord results despite placental dysfunction resulting in trimester-specific antepartum brain lesions with neurologic sequalae. Antepartum placental/cord evaluations using more advanced structural and functional imaging techniques will enhance diagnostic accuracy when compared with postnatal pathological examination [56].

## 7. Pediatric Neurology Evaluations after HDP

During the first two years of life, the expression of developmental disorders and epilepsies [57,58] may be the first opportunity to consider associations with HDP. These negative outcomes occur, despite seemingly normal pregnancy and neonatal time-periods. Prenatal involvement of the FNN when HDP is identified by a high-risk MFM service offers the opportunity to anticipate those MFM triads and neonatal survivors who may be at risk of sequelae, even without the expression of the GOS or GNNS. Future anticipatory neurotherapeutic interventions during prenatal life may avoid or mitigate the sequelae.

Children who experienced the GOS and GNNS after diseases such as HDP more likely require earlier medical interventions at older ages. Hospitalizations, may require pediatric neurointensive care [59] for a more medically-fragile subset. These neonatal critical care survivors more likely require one or multiple pediatric ICU admissions [60,61]. These children more often experience greater numbers and severities of intercurrent illnesses, resulting in more severe neurologic sequelae.

Pediatric neurology consultations in the clinic or hospital settings may originate from primary-care practitioner or pediatric subspecialist referrals when communicable and noncommunicable diseases worsen neurologic function associated with organ-specific systemic diseases. Congenital heart disease (CHD) is the most common anomalous organ system disease of childhood that can be associated with HDP, with sequelae including cognitive/psychiatric disorders [62]. Continuity of risk or cumulative injuries result from medical/surgical interventions and illnesses before and after pregnancy for children with CHD. These associations are pertinent whether affecting a single organ or multi-systemic diseases.

## 8. Childhood, Adolescent and Adult Sequelae

Involvement during the first 1000 days facilitates continued pediatric neurology consultative input throughout childhood into adolescence. This continuity of care more effectively addresses ongoing or new conditions affecting brain health when clinical disorders are expressed in association with HDP. Clinical expression requires brain maturation.

Epileptic, behavioral/cognitive and mental health disorders [58] are associated with HDP. Specific diagnoses, such as attention hyperactivity disorders, learning deficits, sleep disorders and generalized or focal epilepsies, each require focused patient-centric evaluations and targeted interventions, starting during the preschool years. School-aged children express behavioral/cognitive sequalae associated with HDP, requiring neuropsychological evaluations, followed by independent educational plans. Coordination of care with mental health providers needs to address anxiety, mood, sleep, and psychotic disorders, which may present or worsen throughout childhood [63,64] into adolescence. 

Neurologic and mental health disorders associated with HDP may only become clinically apparent with maturation into adulthood [65]. These sequelae may be expressed after acute infectious or inflammatory illnesses, craniocerebral trauma, worsening systemic diseases or adverse environmental exposure. Adult clinical diseases expressed into old age may also be superimposed in earlier neurologic maldevelopment or injury that has already occurred during the first 1000 days. 

Combined developmental origins and life-course approaches to diagnosis and prognosis therefore stress the relevance of the first 1000 days to neurologic/psychiatric diseases presenting across the life-span. Cerebrovascular, cognitive, epileptic and neurodegenerative disorders [1,3] into old age have suggested associations with HDP. Transition to adult neurologic care presents particular challenges for survivors with documented developmental disorders, intellectual disabilities, and pharmaco-resistant epilepsies earlier in life. Greater morbidities and earlier death from the effects of chronic medical illnesses and accidents need to be anticipated [66,67]. This requires a more universal healthcare system across the lifespan, providing accurate documentation. This developmental approach offers a guidepost for the continuity of healthcare throughout a person’s lifespan with adverse effects that will influence future offspring across generations.

## 9. Current Practice Guidelines and Therapeutic Research Advances

Current practice guidelines and stress conventional management strategies to control HDP [68]. Pregnancy-related interventions more commonly are instituted after the 20th week of pregnancy when pre-eclampsia or gestational hypertension are more readily identified based on present standard clinical and laboratory testing [69]. Conventional therapeutic treatments include aspirin and anti-hypertensive medications, often initiated during the latter half of pregnancy. For more late-pregnancy diseases expressed as severe PE, eclampsia and HELLP syndrome, more emergent rescue therapies may be needed, with novel approaches being investigated [70]. For more clinically significant diseases prior to conception or during the first half of pregnancy, conventional treatments must also be considered. However, novel therapeutic interventions need to be investigated for clinical applications [71] to more effectively design neuroprotection.

Adverse effects by HDP on embryonic and early fetal brain development within the MPF triad requires neurotherapeutic interventions, targeted to immature neuronal populations and connectivities that may be impaired during the first half of pregnancy. Pre-conception testing will target inherited genomic biomarkers associated with hypertensive disorders affecting a future pregnancy [12]. The next generation of treatment options for HDP will require applications based on knowledge from the system-biology approach when considering adverse G × E interactions affecting the MPF triad as the pregnancy progresses. Early pregnancy testing should adjust to mitotic and post-mitotic genomic changes that result from the abnormal G × E interactions, as HDP-related diseases appear or worsen. Serial tests of placental reactivity with worsening of HDP are being investigated [72,73]. New placental imaging techniques are being investigated which will have important clinical applications [74] to HDP. Multi-modal testing is needed which combines genetic/metabolic testing compared with fetal placental/neuroimaging/neurophysiologic phenotypes [75,76,77]. Diagnostic approaches, for example need to combine biomarkers such as placental function with volumetric and functional imaging techniques [22] relevant to women expressing HDP.

## 10. Reduction of the Global Burden of Disease

Reduced burden of neurologic disorders across the lifespan from conditions associated with HDP should constitute one of multiple sustainable goals required for maternal and pediatric health initiatives [8]. Significant pediatric population percentages contribute to this global burden, as measured by disability-adjusted life years [78]. Medical interventions for the present and next generations affected by HDP must better document changes in plasticity, growth, neurocognitive development and later development of non-communicable diseases at various stages of the life course These are public health priorities that define a nation’s health and wealth, and effect successive generations [79].

Improvements in both person-centric and population-based healthcare define a nation’s medical, economic and social well-being. Cooperation between government, industry, and nonprofit entities collectively strengthen healthcare policy for populations specific to resource-rich and poor nations. This advocacy must include medical deserts within resource-rich nations. Health disparity research augments efforts to address prenatal and early childhood brain disorders in association with HDP required by public health programs. This is exemplified by the WHO Millennium sustainable goals (i.e., Every Newborn Action Plan), which can more effectively reduce disease burden and financial costs, as well as elevate the quality of life [9]. The significant global incidence of HDP in women during reproductive years highlights the public health priority for this vulnerable pediatric population, with ramification s into adulthood for both older women and their adult offspring.

Interventions need to be offered that are gender-specific and sensitive to sexual-orientation, specifically recognizing the unique aspects of healthcare relevant to women and men [80]. Outcome research for women include ages during and beyond their reproductive years when developing interventions that will improve their multi-systemic health. Neurotherapeutics offered during the first 1000 days will contribute to improved scholastic success, employability and quality of life into old age for mothers and their offspring affected by HDP.

## Abbreviations

HDP	hypertensive disorders of pregnancy
G × E	gene/environment interactions
PE	pre-eclampsia
MPF	maternal/placental/fetal
FNN	fetal neonatal neurology
MIA	maternal immune activation
IPS	ischemic placental syndrome
GOS	great obstetrical syndromes
GNNS	great neonatal neurological syndromes
NE	neonatal encephalopathy
NS	neonatal seizures
NSK	neonatal stroke
EP	encephalopathy of prematurity
